# Comparative evaluation of sump drainage by trocar puncture, percutaneous catheter drainage versus operative drainage in the treatment of Intra-abdominal abscesses: a retrospective controlled study

**DOI:** 10.1186/s12893-015-0049-6

**Published:** 2015-05-09

**Authors:** Guosheng Gu, Jianan Ren, Song Liu, Guanwei Li, Yujie Yuan, Jun Chen, Gang Han, Huajian Ren, Zhiwu Hong, Dongsheng Yan, Xiuwen Wu, Ning Li, Jieshou Li

**Affiliations:** Department of Surgery, Jinling Hospital, Medical School of Nanjing University, 305 East Zhongshan Road, Nanjing, 210002 China; Center for the Study of Inflammatory Bowel Disease, Harvard Medical School, Massachusetts General Hospital, Boston, MA USA; Department of Gastrointestinal-Pancreatic Surgery, the First Affiliated Hospital of Sun Yat-sen University, Guangzhou, China; Department of General Surgery, Second Affiliated Hospital of Jilin University General Surgery, Center of Jilin University, Changchun, China

**Keywords:** Percutaneous catheter drainage, Operative drainage, Sump drainage, Trocar puncture, Intra-abdominal abscesses

## Abstract

**Background:**

Intra-abdominal and pelvic abscesses are common and result from various illnesses. Percutaneous drainage applies limitedly to well-localized abscesses with appropriate density while surgical drainage usually causes significant physiological disturbance. We herein illustrated an innovative choice “sump drainage with trocar puncture” for the management of intra-abdominal abscesses and compare it with conventional percutaneous and surgical drainage in terms of clinical outcomes and prognosis.

**Methods:**

Medical records of a total of 75 patients with abscesses were retrospectively retrieved and scrutinized. Data consisted of demographics, abscesses characteristics and treatment outcomes including postoperative complication, duration of hospitalization, postoperative recurrence of abscesses, subsequent surgery, ultimate stoma creation and survival rate. All enrolled patients were divided into trocar group (n = 30), percutaneous group (n = 20) and surgical group (n = 25) according to the therapeutic modalities. One-way ANOVA and *t*-test with Welch’s correction were used in continuous variables, and Chi-squared test as well as Fisher’s exact test for categorical variables. The cumulative incidence of subsequent surgery and ultimate stoma creation was also indicated by the Kaplan–Meier method and compared by log-rank test.

**Results:**

The risk of ultimate stoma creation (p = 0.0069) and duration of postoperative hospitalization (p = 0.0077) were significantly decreased in trocar group compared with the surgical group. Patients receiving trocar puncture also tended to be less likely to have subsequent surgery (p = 0.097). Patients in trocar group displayed a lower rate of postoperative complication than the percutaneous (p = 0.0317) and surgical groups (p = 0.0175). As for Kaplan–Meier analysis, the cumulative incidence of ultimate stoma creation of the patients using sump drainage was also significantly different among three groups during follow-up period (p = 0.011).

**Conclusion:**

This novel technique “sump drainage by trocar puncture” could produce better clinical outcomes and prognosis than conventional percutaneous drainage and surgical intervention. It might become an optimal choice in the management of intra-abdominal abscesses in the future.

## Background

Intra-abdominal and pelvic abscesses are common and result from various illnesses, such as trauma, surgery and Crohn’s disease [1]. It remains a challenge to clinical doctors as it may result in severe and complicated intra-abdominal and systemic infection together with a high recurrence rate.

Over the last several decades, percutaneous catheter drainage (PCD) has been recommended as a primary choice for source control, and surgical intervention is required for percutaneously inaccessible abscesses or abscesses complicated with perforated diseased bowel [2]. However, the relatively narrow catheter that can only provide simple suction without irrigation function in conventional PCD technique dramatically hampers its application in clinical practice. In most cases, patients with viscous pus, cellulitis, necrotic tissues and especially polycystic abscesses have to receive laparotomy. Surgery is apparently the most direct therapeutic measure to control intra-abdominal and pelvic abscesses, the procedure of which depends on the anatomical source of infection, the degree of peritoneal inflammation, the generalized septic response and the patients’ general conditions [3]. However, the surgical treatment is far more invasive that leads to the more physiologic upset for the patients, and appears to have a higher mortality rate in contrast with the percutaneous approach [4].

To solve this problem, we advocated an innovative device named “sump drain” which is placed under the guidance of trocar puncture to replace the traditional small-diameter catheter in PCD method. Our previous studies [5,6] have reported that this technique providing continuous irrigation and suction function was scarcely blocked by tissues, pus or coagulates and therefore appropriate for the treatment of intra-abdominal abscesses, especially those abscesses that are contraindications for PCD technique.

This study was designed to compare clinical outcomes and prognosis between sump drainage by trocar puncture, conventional PCD and surgical drainage (SD) in the management of intra-abdominal abscesses.

## Methods

### Participants and definitions

We retrospectively collected all patients registered in our unit between January 2006 and February 2012. Patients who were diagnosed having an intra-abdominal or pelvic abscess would be included in this study. The definition of intra-abdominal and pelvic abscesses was extraluminal enhancing fluid collections 1 cm or greater in diameter confirmed by CT scan or ultrasound, with purulence documented at the time of drainage [7]. The exclusion criteria consisted of no treatment of intra-abdominal or pelvic abscess, perianal abscesses and less than 3 months of follow-up period.

In current study, we classified the cause of all abscesses as four main types. 1) Trauma: abscess appeared immediately after trauma or was discovered by CT or ultrasound before operation. 2) Surgery: abscesses emerged within 1 month after surgery that could be due to tumor, obstruction or even trauma. 3) Crohn’s disease and 4) Intestinal tuberculosis: patients who were diagnosed as Crohn’s disease/intestinal tuberculosis and never underwent trauma and surgery would be considered into this category.

All enrolled patients were from the same medical care team in our institution. All operations, including trocar puncture, PCD and SD, were performed by the same surgeon. Prior to the procedures, the risks and benefits of each treatment were discussed with every patient, and informed consent was obtained.

### Data collection

For each participant, we collected the following variables: age, sex, APACHE II score before treatment of abscesses, date of treatment, abscess size and location measured by CT scan or ultrasound, date of discharge and bacteria culture result of purulence samples. The abscess size was determined by the maximum diameter of the abscess.

Our primary outcome was the rate of subsequent surgery and ultimate stoma creation. Secondary endpoints were postoperative complication, duration of hospitalization, recurrence of abscess, and survival condition. The subsequent surgery only included abdominal surgery after abscess treatment and excluded stoma reversal operation. The postoperative recurrence of abscess was defined as the development of an enhancing fluid collection at the same site as the initial lesion occurring within 1 month after the treatment of abscess. Postoperative recurrence of abscess was considered as a separate parameter, instead of being included in postoperative complications.

### Management

For all patients, broad-spectrum antibiotics were prescribed on diagnosis of abscesses and adjusted subsequently according to the bacteria culture result of purulence samples. As a first line therapy in our institution, enteral nutrition was given initially for the majority of enrolled patients with the normal physiological function of the bowel. For patients who failed to tolerate enteral nutrition or suffered intestinal dysfunction, parenteral nutrition was prescribed temporarily. Enteral nutrition would be tried again when the patients’ condition improved, and be established eventually [8].

In the current study, three strategies were used in the management of abscesses. The PCD was defined as procedure using an aspiration and insertion of a conventional drainage catheter. Briefly speaking, after local anesthesia the collection was punctuated with an 18-gauge sheath needle using ultrasound or CT guidance. Then a guidewire was inserted and an 8-F to 16-F pigtail catheter was advanced and sutured to the skin. The size of the catheter depends on the maximum transverse diameter of the abscesses.

The SD was defined as operation with abscess drainage or for exploration, with or without concomitant bowel resection [9,10]. Surgical options included debridement of necrotic tissue, resection or suture of a diseased or perforated viscus, resection of ischemic bowel and repair/resection of traumatic perforations. Laparotomy was usually performed through a midline incision and abscesses cavities were routinely evacuated prior to closure of the fascia. The objectives were both to establish the cause of peritonitis and to control the origin of infection.

The trocar puncture with sump drain was an innovative technique that has been described in our previous studies [5,6]. A specific device named “sump drain” was used to replace the conventional catheter. This sump drain contains a two-channel drainage. The inner tube of the two-channel has vacuum aspiration, while the outer tube is devised with several holes, thereby providing continuous irrigation and suction function. The general procedures are outlined below.

After evaluating the location and depth of the abscess by CT scan or ultrasound, local anesthesia, skin incision (usually 10-15 mm) and blunt dissection of subcutaneous tissues were performed consecutively. Afterwards, a 22-gauge needle was punctured for diagnostic localization and aspiration. A 12-mm laparoscopic trocar was then placed into the abscess cavity through the same direction as the needle. A 10-mm sump drain, which was actually a modified closed double-lumen irrigation-suction tube, was inserted through the trocar cannula and then the trocar was removed, leaving the sump drain in situ.

### Statistics

SPSS software (SPSS for Windows, version 19.0, SPSS, Chicago, IL) was used for all statistical analysis. All analysis was two-tailed and p-values < 0.05 was considered statistically significant. For continuous variables, mean ± SD was calculated. One-way ANOVA was performed to compare variance among three groups and students’ *t*-test with Welch’s correction was used to compare two groups. For categorical variables, chi-squared test was performed to compare the constituent ratio among the three groups, and Fisher’s exact test was performed between two groups. Given the time-dependent property of the probabilities of subsequent surgery and ultimate stoma creation, the cumulative incidence was also calculated (one minus the estimate of the survival function) by the Kaplan–Meier method and compared by log-rank test.

### Ethics

This study was approved by the Ethics Committee of Jinling Hospital.

## Results

A total of 75 patients were enrolled and divided into three groups: 30 patients in trocar group, 20 in PCD group and 25 in SD group. The average age was 42.1 ± 15.9, 36.0 ± 15.5 and 41.2 ± 17.7 years, respectively (p = 0.504). A male predominance was observed in all groups (21 of 30, 11 of 20 and 20 of 25, p = 0.194), which was in accordance with our previous epidemiological study^10^. APACHE II score demonstrated a similar disease severity among patients in all groups (9.50 ± 6.23 for trocar group, 9.06 ± 3.72 for PCD group and 10.0 ± 5.25 for SD group, p = 0.842). In trocar and PCD groups, surgery was the primary cause of abscesses (15 of 30 and 8 of 20, respectively) while Crohn’s disease was the main reason in SD group (11 of 25). The distribution of causes was showed in Table 1.

**Table 1:** Demographics and clinical parameters of patients with intra-abdominal abscesses

	Trocar group (n = 30)	PCD group (n = 20)	SD group (n = 25)	P-value
Age (yr.)	42.1 ± 15.9	36.0 ± 15.5	41.2 ± 17.7	0.504
Male	21	11	20	0.194
APACHE II score	9.50 ± 6.23	9.06 ± 3.72	10.0 ± 5.25	0.842
Cause				0.372
Trauma	9	5	5	-
Surgery	15	8	8	-
Crohn’s disease	5	5	11	-
Intestinal tuberculosis	1	0	1	
Others	0	2^**a**^	0	-

^**a**^pancreatic cancer and B-cell lymphoma

The abscess size was similar among all three groups (7.25 ± 3.63 cm in trocar group, 7.56 ± 3.55 cm in PCD group and 7.00 ± 4.04 cm in SD group, p = 0.754). Abdominal and pelvic cavities were the most common locations for abscess development in all groups. Psoas and intestinal loop were also predilection sites for abscesses. Multiple abscesses were found in 4, 2 and 2 patients in three groups, respectively (Table 2).

**Table 2:** Characteristics of abscesses

	Trocar group (n = 30)	PCD group (n = 20)	SD group (n = 25)	P-value
Abscess size (cm)	7.25 ± 3.63	7.56 ± 3.55	7.00 ± 4.04	0.754
Abscess location				0.980
Psoas	1	1	2	-
Interloop	1	1	2	-
Abdominal	19	14	16	-
Pelvic	5	2	3	-
Multiple abscesses	4	2	2	-
Bacteria of purulence				0.985
Escherichia coli	13	9	14	-
Klebsiella pneumoniae	8	4	5	-
Staphylococci aureus	4	3	2	-
Proteus mirabilis	1	2	1	-
Multiple	2	1	1	-
Others	2^**a**^	1^**b**^	2^**c**^	-

^**a**^Candida albicans and Bacteroides fragilis. ^**b**^Enterococcus faecalis ^**c**^Enterococcus faecalis and Serratia marcescens

13 samples in trocar group, 9 in PCD group and 14 in SD group were demonstrated to contain Escherichia coli in purulence. Klebsiella pneumoniae followed by Staphylococci aureus were also cultured commonly in trocar, PCD and SD groups. Multiple bacteria were found in 2, 1 and 1 patients in three groups, respectively (Table 2).

The average follow-up period for patients in Trocar, PCD and SD groups was 20.7 ± 12.5 months, 14.0 ± 5.16 months and 19.1 ± 8.00 months, respectively (p = 0.0525). Only 1 patient (3.33 %) had postoperative complication (pulmonary infection) in trocar group whereas 5 patients (25.0 %; 1 bleeding, 2 wound infection and 2 pulmonary infection) in PCD and 7 patients (28.0 %; 2 bleeding, 3 wound infection, 1 pulmonary infection and 1 intra-abdominal infection) in SD group had postoperative complications. Statistical analysis suggested a significantly decreased incidence of postoperative complication in trocar group than PCD (p = 0.0317) and SD groups (p = 0.0175). However, statistical difference was not observed between PCD and SD groups (p = 0.999).

**Fig. 1:**
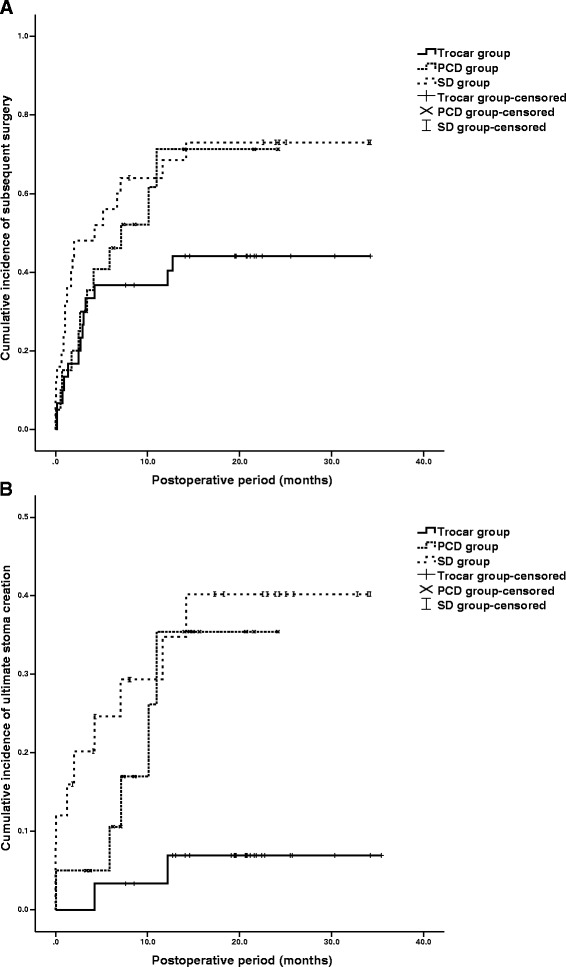
Cumulative incidence of subsequent surgery among the patients in Trocar, PCD and SD groups (**A**). Patients in Trocar group tended to have a lower incidence of subsequent surgery during follow-up period (p = 0.070). Cumulative incidence of ultimate stoma creation among the patients in Trocar, PCD and SD groups (**B**). The cumulative incidence of ultimate stoma creation of the patients receiving trocar puncture was significantly different among three groups during follow-up period (p = 0.011). Cumulative incidence is one minus the estimate of the survival function

Clinical outcomes were indicated in Table 3. In trocar group, 2 patients (6.67 %) had ultimate stoma creation. However, the rate increased up to 25.0 % (5 patients) and 40.0 % (10 patients) in PCD and SD groups. The risk of ultimate stoma creation was significantly decreased in trocar group compared with the surgical group (p = 0.0069). 13 patients (43.3 %) in trocar group whereas 12 (60.0 %) and 18 (72.0 %) in PCD and SD groups underwent subsequent surgery. Lesion resection and anastomosis was the most common type of subsequent surgery for patients with trocar puncture, and stoma creation was the most common approach for patients with PCD and surgical interventions. Patients in Trocar group tended to be less likely to have subsequent surgery (p = 0.097). As for Kaplan–Meier analysis (Fig. 1), the cumulative incidence of ultimate stoma creation of the patients receiving trocar puncture was also significantly different among three groups during follow-up period (p = 0.011).

**Table 3:** Clinical outcomes of management of intra-abdominal abscesses

	Trocar group (n = 30)	PCD group (n = 20)	SD group (n = 25)	p-value
Follow-up period (month)	20.7 ± 12.5	14.0 ± 5.16	19.1 ± 8.00	0.0525
Postoperative complication	1 (3.33 %)	5 (25.0 %)	7 (28.0 %)	**0.0316** ^**a**^
Bleeding	0	1	2	
Wound infection	0	2	3	
Pulmonary infection	1	2	1	
Intra-abdominal infection	0	0	1	
Duration of postoperative hospitalization	15.3 ± 15.2	23.1 ± 24.8	55.4 ± 75.0	**<0.0001** ^**b**^
Postoperative recurrence of abscess	17 (56.7 %)	15 (75.0 %)	15 (60.0 %)	0.399
Subsequent surgery	13 (43.3 %)	12 (60.0 %)	18 (72.0 %)	0.097
Lesion resection and anastomosis	5	1	6	-
Stoma creation	2	5	9	-
Right hemicolectomy	2	4	2	-
Peritoneal lavage	3	2	0	-
Left hemicolectomy	1	0	0	-
Subtotal colectomy	0	0	1	
Ultimate stoma creation	2 (6.67 %)	5 (25.0 %)	10 (40.0 %)	**0.0127** ^**c**^
Death	2 (6.67 %)	3 (15.0 %)	2 (8.00 %)	0.588

^**a**^Trocar *vs.* PCD, **p = 0.0317**; Trocar *vs.* SD, **p = 0.0175**; PCD *vs.* SD, p = 0.999

^**b**^Trocar *vs.* PCD, p = 0.199; Trocar *vs.* SD, **p = 0.0077**; PCD *vs.* SD, **p = 0.033**

^**c**^Trocar *vs.* PCD, p = 0.100; Trocar *vs.* SD, **p = 0.0069**; PCD *vs.* SD, p = 0.352

The duration of postoperative hospitalization was 15.3 ± 15.2, 23.1 ± 24.8 and 55.4 ± 75.0 days in trocar, PCD and SD groups respectively. A remarkable decrease of postoperative hospital stay existed in trocar group compared with SD group (p = 0.0077). Meanwhile, the duration of postoperative hospitalization in PCD group was also shorter than that in SD group (p = 0.033). Patients using trocar puncture did not have statistically different postoperative duration compared with patients using PCD technique (p = 0.199).

17 patients (56.7 %) had postoperative recurrence of abscesses in trocar group while 15 (75.0 %) in PCD group and 15 (60.0 %) in SD group. The difference of postoperative recurrence had no statistical significance (p = 0.399).

During the follow-up period, 2 patients (6.67 %) died in trocar group while 3 (15.0 %) and 2 (8.0 %) died in PCD and SD groups, respectively. Survival rate was similar among all three groups (p = 0.588).

## Discussion

In the current study, we evaluated the clinical outcomes and prognosis of three different strategies in the management of intra-abdominal abscesses. Based on the analysis of 75 eligible patients, our preliminary results suggested several advantages when performing trocar puncture with sump drain instead of traditional PCD and SD. These advantages included decreased rates of ultimate stoma creation and postoperative complication as well as shorter duration of postoperative hospitalization. Moreover, patients with sump drain also tended to have a lower rate of subsequent surgery.

In recent decades, PCD has emerged as a primary option in the management of intra-abdominal abscesses. In the guideline published by Surgical Infection Society and the Infectious Disease Society of America [11], PCD is recommended and preferable to SD in well-localized fluid collection of appropriate density, results in significantly less physiological alteration and reduces the need for open procedure [2]. Open surgical technique is typically considered for poorly localized, complex, or diffuse fluid collections, necrotic tissue, high-density fluid, or percutaneously inaccessible collections. Also, suspicious perforation of diseased bowel with signs of extensive or massive free air requires a laparotomy [2].

Therefore, we can conclude to some extent that there are several hardly-avoidable disadvantages for both PCD and SD. PCD can only be performed on well-localized and low-density abscesses [12,13] while SD may result in a remarkable physiologic alteration, prolonged hospitalization and decelerated recovery process [14,15].

As an innovative drainage technique, trocar puncture with sump drain is being widely used in our clinical practice and has been illustrated in our previous studies [5,6]. It has also been confirmed as a promising management option in Crohn’s disease patients with lower rate of postoperative complication, shorter duration of postoperative hospitalization and lower rate of ultimate stoma creation (our unpublished data), similar to the results of the present study.

This modified technique uses a larger-diameter tube (sump drain) instead of conventional small size catheter to achieve less blockage and to more rapidly obliterate abscess cavity under the assistance of continuous irrigation and suction function with negative pressure. Moreover, this “two-channel tube” device contains an interval at the ends between inner and outer tubes and several holes in the outer tube, which may further reduce the possibility of blockage by tissues, pus or coagulates. Trocar is employed to assist the sump drain reaching the abscess cavity through percutaneous puncture in order to maintain physiologic stability. As a result, this modified technique can be successfully applied to the drainage of viscous pus, cellulitis, necrotic tissues and especially polycystic abscess that are contraindications of PCD [5].

Based on the findings, we conclude that this modified technique not only expands its indications in clinical application in contrast with PCD, but also achieves better clinical outcomes and prognosis compared with SD.

We admit several limitations in the current study. First of all, as a retrospective controlled study, a potential selection bias may exist. However, patients were assigned into three groups after discussing with the surgeons on the risks and benefits of each treatment and signing consent forms. No specific patients were selected to a certain strategy of drainage in the setting of treatment in our center. As the demographic (such as age and gender distribution), clinical (such as APACHE II score and primary cause) and abscess-related parameters (such as abscess size, location and bacteria of purulence) at baseline were all similar among all groups, we assumed these enrolled patients had been relatively well-matched in this controlled study. Second, only 75 qualified patients were enrolled in this study. The small sample size may hinder the statistical power and conceal the results. Besides, a longer follow-up period is also required in larger and multicenter studies.

## Conclusions

In conclusion, we demonstrated that patients with sump drain had lower rates of ultimate stoma creation and postoperative complication as well as shorter duration of postoperative hospitalization compared with those using PCD or SD. This innovative technique may emerge as an optimal choice in the management of intra-abdominal abscesses.

## Abbreviations

PCD: Percutaneous catheter drainage
SD: Surgical drainage
